# Improved Methods of Treatment in Joint Diseases

**Published:** 1863

**Authors:** E. Andrews

**Affiliations:** Professor of Surgery


### IMPROVED METHODS OF TREATMENT IN JOINT DISEASES.

By E. ANDREWS, M.D., Professor of Surgery.

GENTLEMEN:—Within the past three years, the improvements in the treatment of joint diseases have outstripped surgical literature, and have produced valuable practical ideas, which as yet are not to be found in our standard text-books. As I desire to keep you informed in all valuable improvements, I have concluded to call your attention to this topic in particular, this morning.

I present you here three patients. One has a hip-disease; one a disease of the knee-joint; and one a curvature of the spine; three diseases which have been the torment and stumbling-block of surgeons through all time, but which are now about to contribute to their honor and renown.

This case of hip-disease does not differ from others which I have often placed before you, except in being in an older patient. In most instances, it is confined to the age of childhood, but in this instance it commenced at the age of sixteen, and has continued till twenty-two. An English surgeon states, that such late cases do not proceed to suppuration and caries; but this one is both suppurating and carious, and will require excision of the head of the femur. To-day, however, I simply wish you to observe the disease of the hip, for the purpose of fixing in your memory the perfect analogy between hip-disease and knee-disease. Chronic inflammation of the knee-joint, unlike that of the hip, occurs, without distinction, at all ages; but, in its causes, course, and treatment, it follows the same laws. A few months ago this knee began to be tender and lame, apparently, in consequence of a slight injury received in the vicinity. From that time to this it has gradually grown worse. At present it displays the following appearances:—The outline of the knee is a little enlarged and not perfectly natural in shape, indicating that the cartilages and bones are hypertrophied. It is not anchylosed, but the motions are much restricted, and the patient habitually carries it in a bent position. This position is characteristic, and is based on instinct. For to maintain the extended position, the vast strength of the *quadriceps femoris* muscle is brought to bear, and, at the same time, the extension tightens the flexors. All this causes an increase of pressure of the inflamed surfaces of the joint upon each other, and a consequent aggravation of the pain. Hence, the patient, instinctively, bends the knee to such a point as he finds, by experience, gives the least amount of suffering.

The external appearances of these cases differ greatly. Some present us with knees in which you can detect scracely the slightest alteration of the shape, while others show great swelling, anchylosis, and atrophy of the muscles of the thigh. A greater or less degree of lameness always exists.

The disease preserves a perfect anology to hip-disease, in its causes, course, and results. It takes its origin in an aplastic diathesis, acted upon by any mechanical irritation. Thus, it may arise, in such a diathesis, from a blow on the knee, from a wrench or strain, from a fracture in the vicinity, or from over-exercise in running, jumping, or even walking. Like hip-disease also, it does not attack rheumatic constitutions; nor have I ever known rheumatism of the knee to degenerate into this disease. The two seem to be opposed to each other, although, they bear so close a resemblence that, in early stages, I have repeatedly seen this disease mistaken and treated for rheumatism.

It is convenient to divide the course of this disease into two stages.

The first is the stage of inflammation merely, and the second is the stage of caries and suppuration.

In the first stage, we have pain in walking, flexion of the knee, and, generally, some deformity. As the case progresses, it shows, by its appearance, whether it merely affects the synovial membrane, or involves also the cartilages and bones. In the first case, there will be very little deformity, but only pain and tenderness. In the other case, (where the cartilages and bones suffer,) the form of the joint becomes notably changed, and its diameter enlarged. Some cases recover spontaneously from this stage, but others proceed to the effusion of plastic lymph and fibrous anchylosis of the joint. At this point, some additional recoveries occur with loss of motion. A large number, however, unfortunately proceed to the second stage of the disease,—that of suppuration and caries. The capsule of the joint bursts, and the pus gushes out and continues to flow until the patient dies of exhaustion, or, in a few instances, recovers by the slow extrusion of all the carious spiculæ of bone. Such is the course of the common knee-joint disease, which you see is, in all main points, identical in nature and results with hip-disease.

There is one point of considerable interest in the pathology of these cases, which I will mention, because, it directly suggests the proper treatment, and that is the following:—The disease, in the outset, is usually limited to one surface of the joint, and only involves the other by the mechanical pressure and friction which its roughened surface produces. I hold in my hand the bones of a knee-joint which I exsected some time since in this disease. Now, observe that the femur would seem to have been the starting-point of the disease, and to have then implicated the bones articulating to it. On the lower surface of the inner condyle of the femur you observe a sequestrum about the size of a dime, lying loosely in its bed, but which could not leave its location and be cast off, because it was capped in and held by the concavity of the tibia on which it rested. Such being the case, the following results were inevitable:—The cartilage upon the face of the sequestrum would rapidly disentegrate and disappear, and the rough naked piece of bone bathed in pus, lifted by the granulations in its bed, would press directly upon the articular surface of the tibia. By continuing this pressure, it would rapidly cause a corresponding ulceration and disintegration of the tibial cartilages; and, when that was removed, a denuding and necrosis of a corresponding spot of the bony articular face of the tibia.

Now, look at these two bones more attentively, and you will observe, that, directly at the point where the sequestrum of the femur pressed, there is a sequestrum in the tibia, so accurately fitted, both in size and location, that the one must have rested exactly upon the other. On the anterior portion of the femoral articular face is another sequestrum, and exactly fitted upon it is another in the posterior surface of the patella. These significant points show that the disease extends from one bone to another, by the simple effect of mechanical pressure and friction of the roughened surfaces against each other; and they prove, therefore, the importance of devising apparatus to take off that pressure and prevent that friction.

The treatment of knee-joint disease is, as already intimated, identical with that of hip-disease, and mainly mechanical. I have often called your attention to the fact, that the reason why inflammations of the hip-joint do not so often recover spontaneously, like those of the shoulder, is, because the inflamed head of the femur is ground upon harshly by the weight of the body in walking. The same principle is *a fortiori* true in the knee-joint. Hence, in the treatment, the first and foremost object is to apply some apparatus which shall effectually take off the weight of the body and the tension of the muscles from the joint. Moved by these reasonings, I have been in the habit of treating the disease in question by the same splint which I use for hip-disease, and with excellent success. I was not aware that any one else had tried the same experiment until a few days since, when I learned that Dr. DAVIS, of New York, the worthy discoverer of the splint treatment for hip-disease, had also, before myself applied similar apparatus to the knee, and with even greater brilliancy of success than in the hip. I consider it thoroughly settled, therefore, that the mechanical treatment is as applicable to the knee as to the hip; and that in both the early application of a suitable splint will, generally, produce a recovery by resolution. In fact, these joints, when freed from mechanical irritation, seem as ready to recover spontaneously from attacks of inflammation as any other portion of the body. I say these things to impress upon you that the mechanical treatment is the main agent in effecting a cure, and that you may rely thoroughly upon its efficiency; but, at the same time, I do not wish you to neglect the general health and the diathesis. The health should be steadily promoted by every influence which is adapted to increase its tone and vigor; but, especially, you should take care, all through the case, that the patient shall never, for a single day, fall into the aplastic diathesis. If he does, he will find it a day of peril, for, if he undergoes the least degree of suppuration or caries, he will lose the joint. You are aware that, in the aplastic diathesis, suppuration and caries follow very moderate inflammations, while, in the plastic condition, they might not occur for years. I advise, therefore, that the patient shall have perfectly fresh air both night and day, never on any account being allowed to lodge or reside in crowded buildings. He should use animal food freely; and, if at any time marks of aplasticity show themselves, such as weak purulent eruptions, erysipelas, or other aplastic diseases, he should be immediately put upon the use of muriated tincture of iron, 40 drops every two hours, which may be assisted by the application of muriatic or sulphuric acid baths daily to the whole body.

It is very common to see blisters and issues recommended for this disease; but, in my experience, I have been able to get little benefit from them, and I have no hesitation in saying, that they are thoroughly uncertain and unreliable. If used at all, you should take care that you do not apply them directly to the joint, but at some distance above or below. I occasionally see considerable mischief done by placing the blisters directly upon the knee. The irritation of the blister is then so close to the disease that it spreads into and aggravates it. It, therefore, ceases to be *counter*-irritant, and becomes an irritation direct.

By this combination of mechanical and constitutional treatment, most of your cases can be cured in the first stage. If, however, one is brought to you, already in the second stage, there is no remedy but amputation or resection of the carious bone. If the patient is poor, and necessitated to get about for his subsistence as soon as possible, perhaps, amputation is the best; but, if his circumstances enable him to give six months or a year to treatment, he may undergo resection, with a view to the saving of the limb.

I perceive that our time will not permit of a discussion of the curvature of the spine to-day, I shall, therefore, reserve that subject for another lecture.

